# The Chromatin Remodeling Factor SMARCB1 Forms a Complex with Human Cytomegalovirus Proteins UL114 and UL44

**DOI:** 10.1371/journal.pone.0034119

**Published:** 2012-03-27

**Authors:** Toril Ranneberg-Nilsen, Halvor Rollag, Ragnhild Slettebakk, Paul Hoff Backe, Øyvind Olsen, Luisa Luna, Magnar Bjørås

**Affiliations:** 1 Department of Microbiology, University of Oslo and Oslo University Hospital HF, Rikshospitalet, Oslo, Norway; 2 Department of Medical Biochemistry, University of Oslo and Oslo University Hospital HF, Rikshospitalet, Oslo, Norway; 3 Centre for Molecular Biology and Neuroscience, University of Oslo and Oslo University Hospital HF, Rikshospitalet, Oslo, Norway; University of Sussex, United Kingdom

## Abstract

**Background:**

Human cytomegalovirus (HCMV) uracil DNA glycosylase, UL114, is required for efficient viral DNA replication. Presumably, UL114 functions as a structural partner to other factors of the DNA-replication machinery and not as a DNA repair protein. UL114 binds UL44 (HCMV processivity factor) and UL54 (HCMV-DNA-polymerase). In the present study we have searched for cellular partners of UL114.

**Methodology/Principal Findings:**

In a yeast two-hybrid screen SMARCB1, a factor of the SWI/SNF chromatin remodeling complex, was found to be an interacting partner of UL114. This interaction was confirmed *in vitro* by co-immunoprecipitation and pull-down. Immunofluorescence microscopy revealed that SMARCB1 along with BRG-1, BAF170 and BAF155, which are the core SWI/SNF components required for efficient chromatin remodeling, were present in virus replication foci 24–48 hours post infection (hpi). Furthermore a direct interaction was also demonstrated for SMARCB1 and UL44.

**Conclusions/Significance:**

The core SWI/SNF factors required for efficient chromatin remodeling are present in the HCMV replication foci throughout infection. The proteins UL44 and UL114 interact with SMARCB1 and may participate in the recruitment of the SWI/SNF complex to the chromatinized virus DNA. Thus, the presence of the SWI/SNF chromatin remodeling complex in replication foci and its association with UL114 and with UL44 might imply its involvement in different DNA transactions.

## Introduction

The human cytomegalovirus (HCMV), a member of the *Betaherpesviridae*, is an important human pathogen. It is the major viral cause of birth defects [1], [2] and represents a major medical problem in immunocompromised individuals, such as AIDS patients and patients with allogenic solid organ or stem cells transplants [3]–[5].

The HCMV-genome is one of the largest human DNA-virus genomes (230 kbp) with about 200 predicted open reading frames [6]. After binding of HCMV to cell surface receptors the virus membrane and cell membrane fuse, and the nucleocapsid is released into the cytoplasm. The nucleocapsid transverses the cytoplasm by association with the microtubules network and gain access to the nuclear pores where the uncoating is completed and the viral genome is released into the nucleoplasm [7]. Immediately after entering the nucleoplasm the viral DNA is circularized, histone proteins bind to virus DNA and nucleosomes are formed [8], [9]. The HCMV genomes serve as templates for transcription and replication, thought to take place within discrete nuclear inclusions. These inclusions develop adjacent to small sites known as promyelocytic leukemia bodies or nuclear domain 10 and take over large parts of the nuclear space at late times post infection [10], [11]. HCMV replication requires a conserved set of six core DNA replication proteins: the DNA polymerase (UL54) and the associated polymerase processivity factor (UL44), a single-stranded DNA binding protein (SSB; UL57), and the triplex containing DNA helicase (UL105), primase (UL70) and primase-associated factor (UL102) subunits [6], [12], [13]. HCMV UL114 encodes a uracil-DNA glycosylase homolog that is highly conserved in all characterized herpesviruses that infect mammals [14]. Analysis of HCMV-DNA replication kinetics performed using a HCMV UL114 deletion mutant has shown that the initial rate of DNA synthesis and the accumulation of progeny viral genomes were significantly reduced compared to the parent virus [15], [16]. UL114 is thus obviously of importance for several steps in HCMV-DNA synthesis.

The assembly and organization of nucleosomes on viral genomes is thought to be linked to the replication mechanisms and life cycle of the virus [17]. Modifications of the chromatin structure on HCMV-DNA are thus of importance both for transcription, replication and regulation of latency [18]–[21]. The chromatin structure on DNA is modified by post-translational modification, e.g. acetylation and methylation of N-terminal tails of histone proteins and by chromatin remodeling [22]–[24]. There are currently four different families of chromatin remodeling complexes; SWI/SNF (switching defective/sucrose non-fermenting), ISWI (imitation switch), CDH (chromo domain helicase DNA-binding) and INO80 (inositol requiring 80) family [23], [25]. These ATP dependent chromatin remodeling complexes work in concert with histone tail modifying enzymes [24].

In order to elucidate the mechanisms of UL114 mediated enhancement of HCMV-DNA-replication we have searched for cellular partners to UL114 by a two-hybrid assay. One of the clones identified by sequencing was the human SMARCB1 protein, a core subunit of the highly conserved multi subunit SWI/SNF chromatin remodeling complex. In this work we characterized the intracellular localization of SMARCB1 throughout infection showing re-localization to the replication foci. Further experiments showed direct interaction of SMARCB1 and UL114 and SMARCB1 and UL44.

## Results

### Yeast two-hybrid experiments identify the cellular factor SMARCB1 as a strong binding partner to UL114

**Figure 1:**
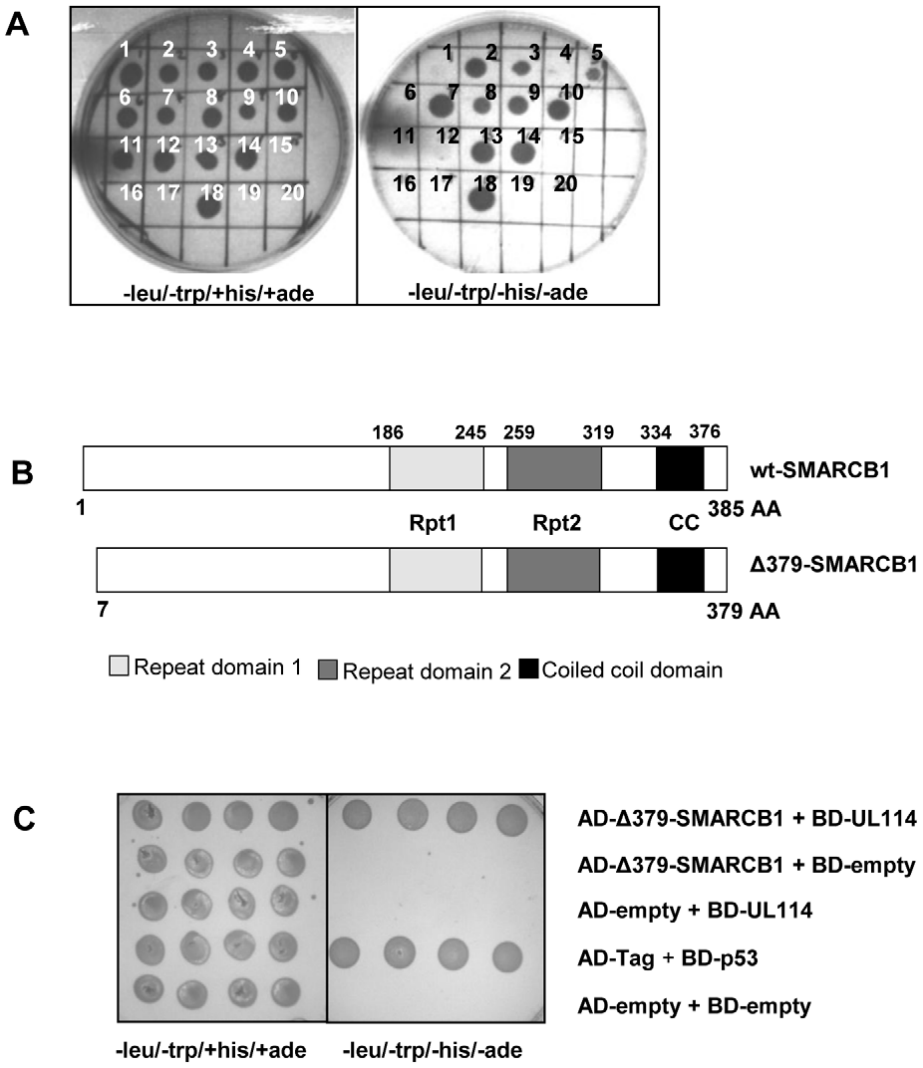
Direct yeast two-hybrid analysis of putative interacting partners of UL114. Two-hybrid screening of a brain cDNA library (activation domain (AD)) using UL114 as a bait (binding domain (BD)) identified several potential interacting cellular partners. (**A**) 17 unique clones were subjected to direct two-hybrid analysis. Self-activation of the reporter genes lacZ and HIS3 was tested for each of the 17 clones by co-transforming and plating each of the putative interacting clones (interactors 1 to 17-AD) and the empty bait plasmid (empty-BD) on selective media: (−leu/−trp/+his/+ade) for cell viability and (−leu/−trp/−his/−ade) for self-activation. Single colonies diluted in water at equal density were spotted onto selective media as indicated. Clones 1–11 and 13–17: interactor 1–11 and 13–17+BD-empty (pGBKT7); clone 12: AD-Δ379-SMARCB1+BD-empty (pGBKT7); clone 18: positive control, AD-Tag+BD-p53. Clones 1, 4, 6, 11 and 12 did not self-activate. (**B**) Schematic presentation of wild type (wt) SMARCB1 and truncated Δ379-SMARCB1 identified by the two-hybrid analysis (clone 12 from Figure 1A). Numbers indicate amino-acid residues. SMARCB1 has two highly conserved domains (Rpt1 and Rpt2) that are imperfect direct repeats of each other and a third conserved coiled coil domain at the C terminus (CC). (**C**) Extensive direct two-hybrid analysis of truncated SMARCB1 (Δ379-SMARCB1). Strong interaction was tested by co-transformation and plating of AD-Δ379-SMARCB1+BD-UL114 on selective media (−leu/−trp/−his/−ade). Self-activation was tested by co-transformation and plating of AD-Δ379-SMARCB1+BD-empty and AD-empty+BD-UL114 on selective media (−leu/−trp/−his/−ade). Four single colonies diluted into water at equal density were spotted onto selective media as indicated. Positive control: AD-Tag+BD-p53, Negative control: AD-empty+BD-empty.

To identify cellular partners of the HCMV encoded uracil DNA glycosylase, UL114, we used a yeast based two-hybrid system with a “bait” plasmid (pGBKT7-UL114) encoding the full-length UL114 protein fused to GAL4 DNA-binding domain (BD) in pGBKT7 transformed into the yeast strain AH109. The two-hybrid screen was performed by mating a yeast strain Y187 pre-transformed with a highly complex brain cDNA library cloned into the yeast GAL4 activation domain (AD) vector pACT2 with AH109-pGBKT7-UL114. Cells were grown on synthetic dropout (SD) medium; SD-trp/-leu/-his/-ade, to select for strong interactions. More than 350 clones were found to grow under these strict conditions indicating several putative cellular partners to UL114. All of these clones were subjected to automated sequencing and homology searches in NCBI databases. Because a large number of clones were screened, several independent clones containing the same binding domain were isolated in many cases. After sorting the sequencing data eliminating typically false positives and taking into account clones represented more than once, 17 clones were subjected to direct two-hybrid analysis (Figure 1A). Self-activating clones were identified by mating the 17 clones with the empty BD vector (pGBKT7) and plating in selective media: −leu/−trp/+his/+ade for cell viability and −leu/−trp/−his/−ade for self-activation (clones 2, 3, 5, 7, 8, 9, 10, 13 and 14; Figure 1A). One of the clones that did not self-activate (clone 12, Figure 1A) was identified as a partial cDNA encoding for human SMARCB1 (SNF5/INI1/BAF47) (379 aa from accessory number NM_003073.2, Gene ID: 6598), termed Δ379-SMARCB1 in this paper (Figure 1B). The specific interaction between Δ379-SMARCB1 and UL114 was confirmed by direct two-hybrid analysis with various controls (Figure 1C).

### SMARCB1 and UL114 interact *in vitro*

**Figure 2:**
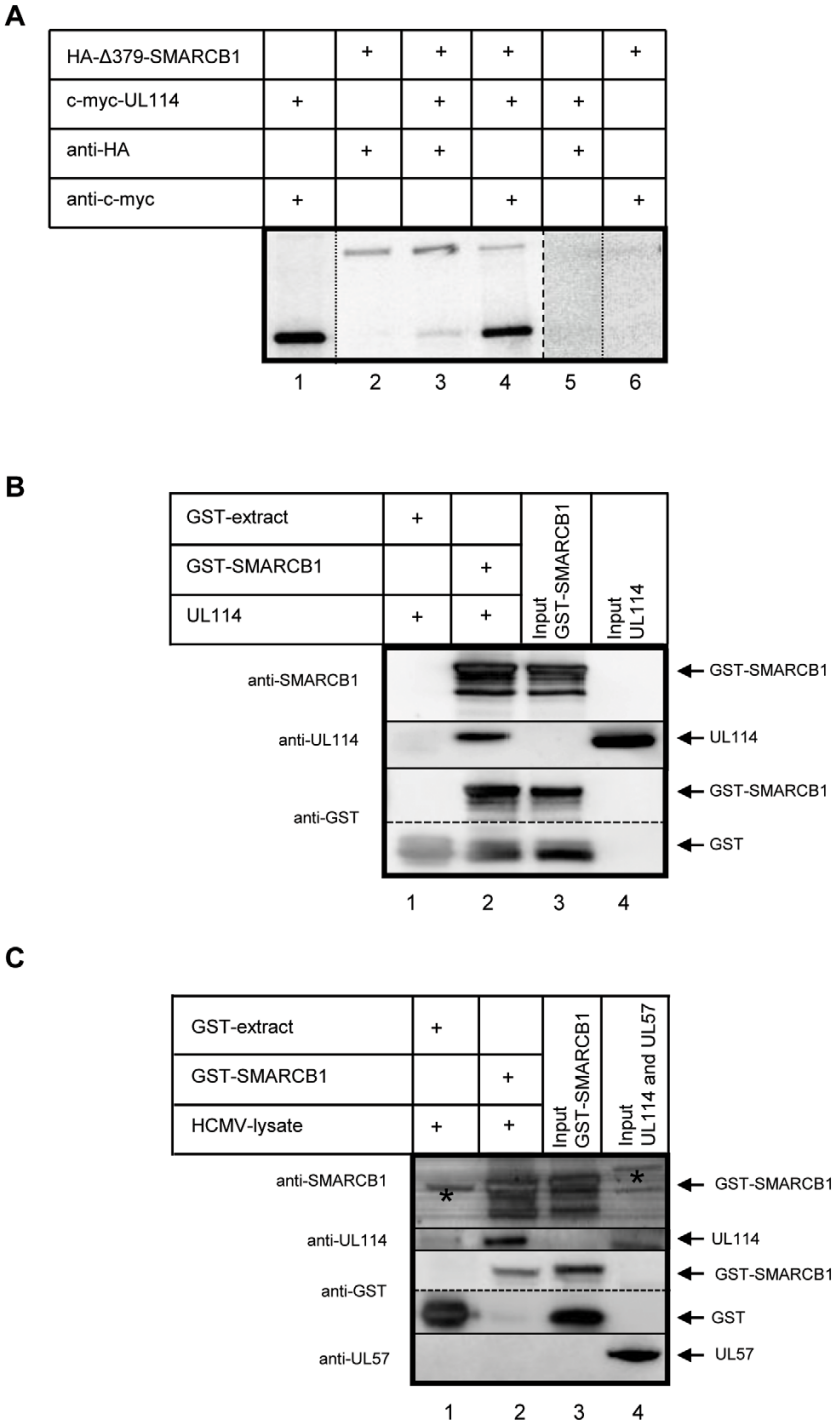
SMARCB1 and UL114 interact *in vitro*. (**A**) *In vitro* binding analysis of HA-tagged Δ379-SMARCB1 and c-myc-tagged UL114 in ^35^S-labeled proteins using the TNT coupled transcription/translation system. The proteins were transcribed and translated *in vitro* with ^35^S-methionine in the translation mixture to generate radioactive labeled products from vectors pACT2-Δ379-SMARCB1 (HA-epitope) and pGBKT7-UL114 (c-myc epitope). The translated Δ379-SMARCB1-HA and UL114-c-myc were immunoprecipitated with either anti-HA or anti-c-myc-antibodies and eluted from the Protein G beads. Samples were subjected to 8% SDS-PAGE and PhosphoImaging. Lane 1: UL114-c-myc+c-myc antibody. Lane 2: Δ379-SMARCB1-HA+HA-antibody. Lane 3: Δ379-SMARCB1-HA+UL114-c-myc+HA-antibody. Lane 4: Δ379-SMARCB1-HA+UL114-c-myc+c-myc antibody. Lane 5: UL114-c-myc+HA-antibody. Lane 6: Δ379-SMARCB1-HA+c-myc-antibody. (^.......^) indicates that samples were run on the same gel and (——) indicates that samples were run on a different gel. (**B**) and (**C**) GST pull-down assays to detect the interaction of SMARCB1 and UL114. (**B**) Purified GST-SMARCB1 or crude extract of *E. coli* cells over-expressing GST were incubated with purified UL114. The GST pull-down products were immunoblotted with anti-SMARCB1, anti-UL114 and anti-GST. Lane 1: GST-extract+UL114. Lane 2: GST-SMARCB1+UL114. Lane 3: purified GST-SMARCB1 (2 µg, 10% of input). Lane 4: Purified UL114 (1 µg, 5% of input). Note that spontaneous cleavage of GST occurred in the GST-SMARCB1 protein sample (Lanes 2 and 3). (**C**) Purified GST-SMARCB1 or crude extract of *E. coli* cells over-expressing GST were incubated with lysates of HCMV-infected cells. The GST pull-down products were immunoblotted with anti-SMARCB1, anti-UL114, anti-GST and anti-UL57. Lane 1: GST-extract+HCMV lysate. Lane 2: GST-SMARCB1+HCMV lysate. Lane 3: purified GST-SMARCB1 (2 µg, 10% of input). Lane 4: HCMV lysate (30 µg, 1% of input). Note that spontaneous cleavage of GST occurred in the GST-SMARCB1 protein sample (Lanes 2 and 3). The asterisks (*) on Lanes 1 and 4 indicates unspecific bands by the use of anti-SMARCB1.

In order to test whether the interaction between UL114 and Δ379-SMARCB1 was reproducible *in vitro*, three sets of experiments were performed. First, by coupled *in vitro* transcription/translation and co-immunoprecipitation (IP), we examined the interaction between the two proteins using [^35^S]methionine-labeled HA-tagged Δ379-SMARCB1 and [^35^S]methionine-labeled c-myc-tagged UL114. IP with anti-HA antibody recovered HA-Δ379-SMARCB1 and c-myc-UL114 (Figure 2A, lane 3). Reciprocally, c-myc-UL114 and HA-Δ379-SMARCB1 were detected when immunoprecipitation was performed with anti-c-myc antibody (Figure 2A, lane 4). Control immunoprecipitations with an irrelevant antibody and protein were carried out to confirm the specificity of the interaction between Δ379-SMARCB1 and UL114. Thus, c-myc-tagged UL114 was not immunoprecipitated by the HA antibody (Figure 2A, lane 5) and HA-tagged Δ379-SMARCB1 was not immunoprecipitated by the c-myc antibody (Figure 2A, lane 6). Moreover, an independent HA-tagged protein HA-clone 4 (clone 4 from Figure 1A) was not immunoprecipitated by c-myc-UL114 (Figure S1).

Second, direct interaction studies between UL114 and SMARCB1 were carried out using a glutathione S-transferase (GST) pull-down assay. To this end, full-length SMARCB1 was cloned into a bacterial GST expression vector and purified. GST was also expressed; however the expression was so high that no purification was needed for these experiments. GST-SMARCB1 or crude GST extract incubated with purified UL114 were subjected to conventional pull-down analysis by the use of glutathione (GST-binding) magnetic beads. After pull-down, the samples were analyzed by western blotting using anti-UL114, anti-SMARCB1 and anti-GST antibodies. Western blot results shown in Figure 2B, indicated that UL114 bound to SMARCB1 directly.

Third, to investigate whether SMARCB1 and UL114 interacted *in vivo*, human fibroblast cells were infected with HCMV. The lysates were immunoprecipitated with anti-SMARCB1 antibodies. Immunoprecipitation with SMARCB1 antibody clearly showed input SMARCB1, however, we could not detect UL114 in the precipitate (data not shown). As previously reported the reverse co-immunoprecipitation experiment with UL114 antibodies was not possible because the available UL114 antibody lacked immunoprecipitation properties [26]. Thus, GST-SMARCB1 and GST immobilized on GST-binding magnetic beads were incubated with HCMV-infected cell lysates. After pull-down, the samples were analyzed by western blotting using anti-UL114, anti-SMARCB1, anti-GST and anti-UL57. As shown in Figure 2C, UL114 from HCMV infected cells was found to interact robustly with GST-SMARCB1 but not to GST alone. The specificity of the assay was tested by blotting against UL57, the single-stranded DNA binding protein. As seen in Figure 2C, UL57 was readily detected in the HCMV lysate (lane 4), however, it was not precipitated with GST-SMARCB1. These *in vitro* binding results confirm the yeast two-hybrid interaction between UL114 and SMARCB1.

### SMARCB1 is recruited to HCMV replication foci

**Figure 3:**
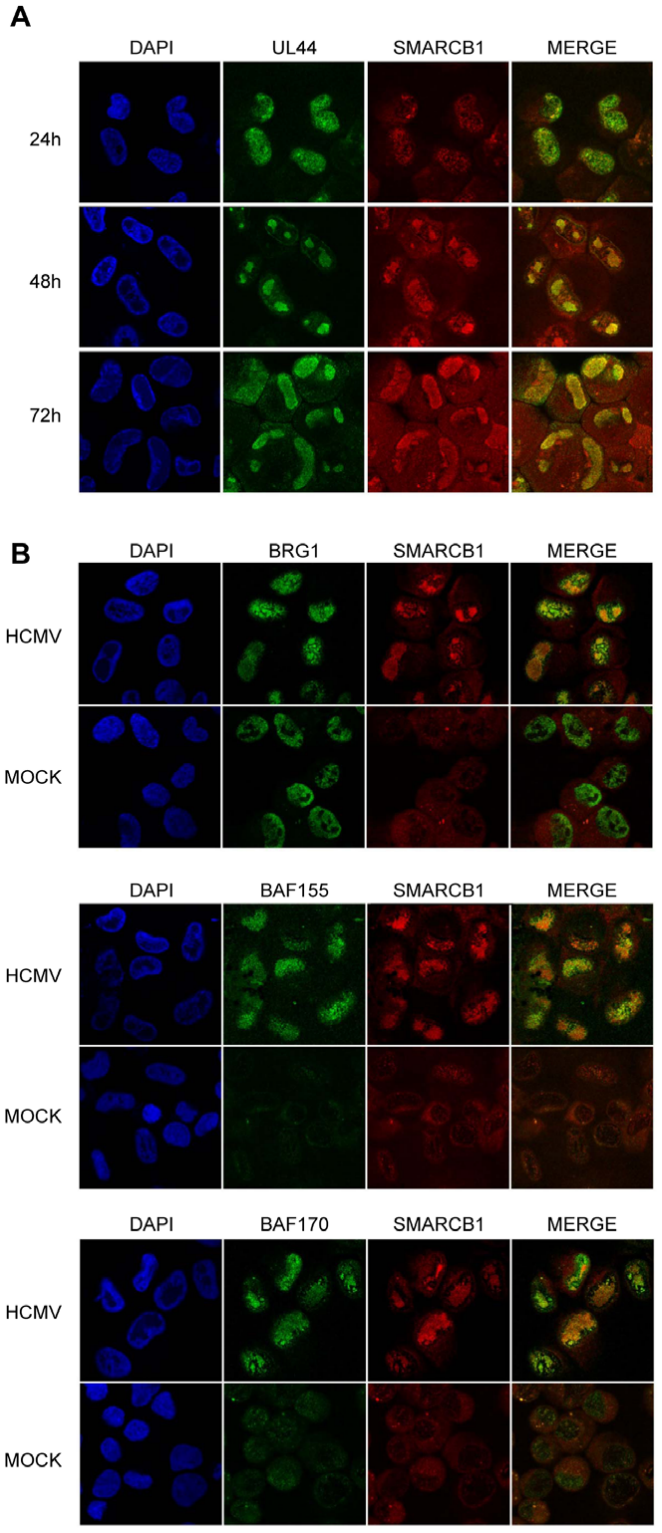
Recruitment of SWI/SNF chromatin remodeling factors to nuclear virus DNA replication foci. (**A**) SMARCB1 co-localizes with UL44 in HCMV-infected fibroblast cells harvested at 24, 48, and 72 hpi. (**B**) Co-localization of SMARCB1 and other essential factors of the SWI/SNF complex; BRG-1, BAF155, BAF170, in HCMV infected fibroblast cells harvested at 48 hpi. The cells were fixed and subjected to double-staining for UL44 (mouse Mab-UL44) and SMARCB1 (rabbit Pab-SMARCB1) and SMARCB1 (rabbit Pab-SMARCB1) and either BRG-1 (mouse Mab-BRG-1), BAF155 (mouse Mab BAF155), BAF170 (mouse Mab BAF170) for immunofluorescence microscopy. Secondary antibodies were used for staining UL44, BRG-1, BAF155, BAF170 in green (anti-mouse 488) and SMARCB1 in red (anti-rabbit 594), and the cells were further visualized by confocal microscopy. Co-localization was visualized by a merge of the two microscopic determinations, and counterstaining of the nuclei was achieved by the use of DAPI.

As described previously the essential viral replication proteins organize into discrete nuclear foci, termed pre-replication sites that mature into viral DNA replication compartments [26], [27]. UL44 is recruited into pre-replication sites already at 5 hours post infection (hpi), prior to the onset of DNA replication and can thus be used as a HCMV replication focus marker [16], [26]. We investigated whether SMARCB1 was recruited to these sites during HCMV infection by assessing intracellular localization of SMARCB1 in mock and HCMV-infected fibroblast cells at immediate –early (5–12 hpi), early (24 hpi) and late (48 and 72 hpi) time points of infection. We showed that SMARCB1 was recruited to viral replication foci in HCMV-infected cells and co-localized with UL44 throughout the infection cycle (24–72 hpi) (Figure 3A). In mock fibroblast cells the localization of SMARCB1 was hampered by high background staining in contrast to HCMV infected fibroblast cells (Figure S2 and Figure 3B). Likewise, attempts to visualize co-localization at immediate –early (5–12 hpi) time points of infection were hampered by high background staining (data not shown). Since only SMARCB1 and UL114 rabbit antibodies functioning in immunofluorescence were available, co-localization studies could not be carried out. However, previous results have shown that UL114 localizes to viral replication foci as early as 5 hpi and co-localizes with UL44 throughout the infection cycle (5–72 hpi) [26], thus indirectly indicating that SMARCB1 and UL114 co-localize.

To investigate whether the entire 2 MDa multi SWI/SNF complex or only the SMARCB1 protein was recruited to the viral replication foci, we examined co-localization between UL44 and three other proteins in the multi SWI/SNF complex; the central ATPase subunit; Bramha-related gene-1 (BRG-1), BAF170 and BAF155, which in addition to SMARCB1 are required for efficient chromatin remodeling activity [28]. As shown in Figure 3B they were all recruited into HCMV replication foci (48 hpi).

### The expression of the core SWI/SNF chromatin remodeling factors increase in HCMV-infected fibroblast cells

**Figure 4:**
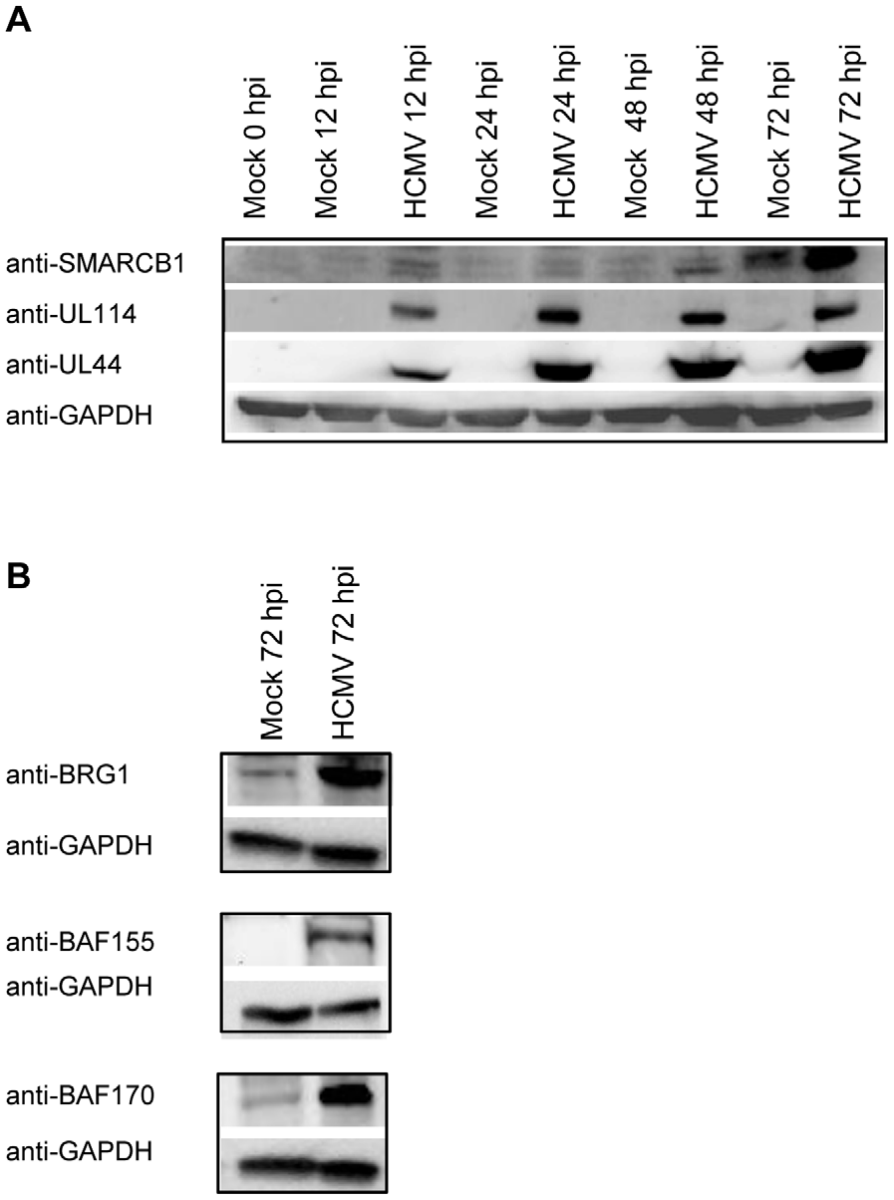
Increased expression of the SWI/SNF core subunits in HCMV infected cells. (**A**) The expression of UL114, SMARCB1 and UL44 in nuclear extracts (20 µg) from mock and HCMV-infected fibroblast cells was analyzed at immediate –early (12 hpi), early (24 hpi) and late (48 and 72 hpi) times of infection by western blot. The western blots were analyzed by Thyphoon scanning. GAPDH was used as a loading control. (**B**) The expression of BRG1, BAF155 and BAF 170 in nuclear extracts (40 µg) from mock and HCMV-infected fibroblast cells was analyzed at late (72 hpi) time of infection by western blot. The western blots were analyzed by Thyphoon scanning. GAPDH was used as a loading control.

The experiments shown in Figure 3 suggested that the expression of SWI/SNF members tested increased during HCMV infection. We first, analyzed the expression of SMARCB1, UL114 and UL44 in nuclear protein extracts at immediate –early (12 hpi), early (24 hpi) and late (48 and 72 hpi) times of infection. Results showed that the expression of SMARCB1 increased in both mock (72 hpi) and HCMV-infected cells (Figure 4A). Also, UL114 and UL44 increased throughout the infection cycle according to previously published data (Figure 4A) [26]. Next we examined nuclear extracts at late (72 hpi) times of infection for the expression of BRG1, BAF 170 and BAF 155. As seen in Figure 4B, all three proteins showed higher expression in HCMV infected cells compared to mock cells.

### SMARCB1 and UL44 interact *in vivo* and *in vitro*

**Figure 5:**
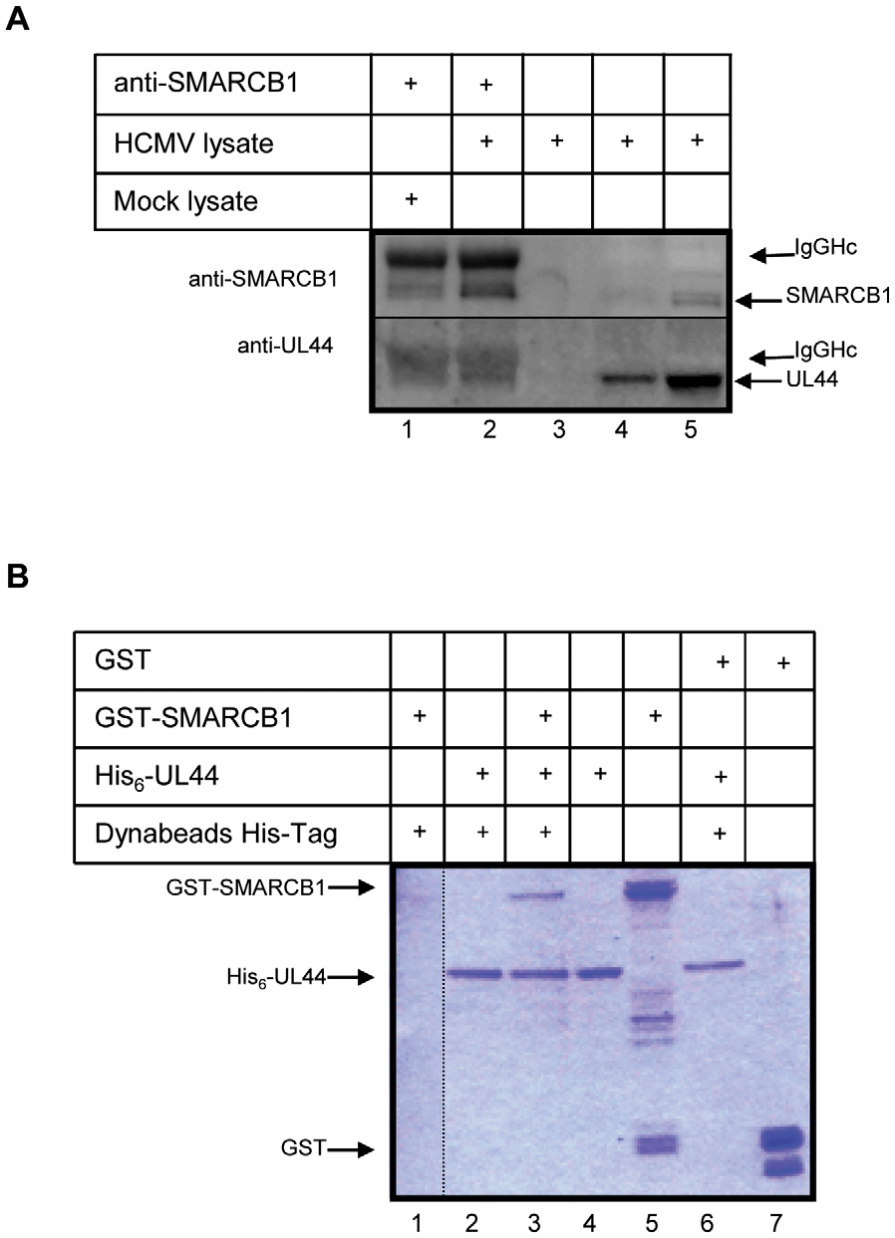
Interaction of SMARCB1 and UL44 in HCMV-infected fibroblast cells and with recombinant proteins. (**A**) Co-immunoprecipitation of endogenous SMARCB1 and UL44 in HCMV-infected fibroblast cells. Equal numbers of mock infected and HCMV infected fibroblast cells were lysed at 72 hpi, and the extracts were immunoprecipitated with anti-SMARCB1. Co-immunoprecipitated proteins were resolved electrophoretically and subjected to immunoblot analysis with anti-UL44 and anti-SMARCB1. Lane 1: Mock lysate immunoprecipitated with anti-SMARCB1. Lane 2: HCMV lysate immunoprecipitated with anti-SMARCB1. Lane 3: HCMV lysate immunoprecipitated with no antibody. Lanes 4 and 5, SMARCB1 and UL44 input in extracts (7 µg, 1% of the total in Lane 4 and 35 µg, 5% of the total in Lane 5). IgGHc: IgG heavy chain. (**B**) *In vitro* pull-down assay of GST-SMARCB1 and His_6_-UL44. Purified GST-SMARCB1 or GST incubated with purified His_6_-UL44 immobilized on magnetic His-tag Dynabeads. Samples were analyzed by SDS-PAGE and Coomassie blue staining. Lane 1: GST-SMARCB1+Dynabeads His-tag. Lane 2: His_6_-UL44+Dynabeads His-tag. Lane 3: GST-SMARCB1+His_6_-UL44+Dynabeads His-tag. Lane 4: His_6_-UL44 (2 µg, 10% of input). Lane 5: GST-SMARCB1 (2 µg, 10% of input). Lane 6: GST+His_6_-UL44+Dynabeads His-tag. Lane 7: GST (2 µg, 10% of input). Note that spontaneous cleavage of GST occurred in the GST-SMARCB1 protein sample (Lane 5). (^……^) indicates that samples were run on the same gel.

As co-localization studies indicated that SMARCB1 was associated with the viral replication apparatus, experiments investigating the interaction between SMARCB1 and UL44 were undertaken. First, we performed experiments in which SMARCB1 was immunoprecipitated with anti-SMARCB1 antibody from cell extracts prepared from HCMV-infected fibroblast cells. Western blot analysis of the immunoprecipitated samples revealed the presence of UL44 (Figure 5A, lane 2). UL44 could not be detected in IP experiments with no antibody or from uninfected cells (mock) (Figure 5A, lanes 3 and 1, respectively). The interaction between SMARCB1 and UL44 could be mediated by UL114, other viral and cellular proteins or DNA. We thus performed an experiment with purified proteins to analyze if it was a direct interaction between SMARCB1 and UL44. UL44 was cloned into a bacterial His_6_ expression vector and purified. A His_6_ pull-down assay was carried out using purified His_6_-UL44 immobilized to magnetic beads with excess of purified GST-SMARCB1 and GST. His_6_-UL44 was able to precipitate GST-SMARCB1 but not GST alone (Figure 5B, lanes 3 and 6, respectively). The specificity of the binding reaction assay was tested using His_6_-NEIL1 instead of His_6_-UL44 as an irrelevant protein (Figure S3). We have thus demonstrated a direct interaction between UL44 and SMARCB1.

### Expression and sub-nuclear distribution of SMARCB1, UL114, and UL44 in the nuclei of HCMV-infected fibroblast cells

**Figure 6:**
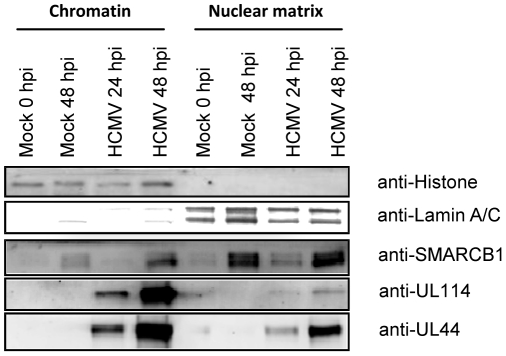
SMARCB1, UL114 and UL44 are associated with the chromatin and the nuclear matrix in HCMV infected fibroblast cells. Mock- and HCMV-infected fibroblast cells harvested at indicated time points were subjected to sub-nuclear fractionation to obtain whole chromatin fraction and core nuclear matrix. Proteins from equal cell equivalents from each fraction were analyzed by western blotting with the indicated antibodies.

Biochemical and genetic evidence suggest that the SWI/SNF complex is involved in the remodeling of chromatin during gene activation [24], [29], [30]. Studies have shown that several components of the SWI/SNF complex are enriched in active chromatin and are associated with the nuclear matrix [31]. We therefore examined the sub-nuclear localization of SMARCB1, UL114 and UL44 in mock and HCMV-infected fibroblasts at early (24 hpi) and late (48) times of infection by biochemical fractionation and western blot analysis. Extracts from mock-and HCMV-infected cells were prepared to obtain the soluble chromatin and the nuclear matrix and analyzed by western blotting. The fractionation procedure was controlled by immunoblotting with antibodies directed against lamin A/C, a nuclear matrix-associated protein, and histone H1, a chromatin-associated protein. Immunoblotting (Figure 6) revealed that SMARCB1, UL114 and UL44 were present in both the soluble chromatin and nuclear matrix fractions of the HCMV-infected cells. Both mock- and HCMV-infected cells showed highest enrichment of SMARCB1 in the nuclear matrix fraction 48 hpi. The UL114 and UL44 proteins showed the highest enrichment in the chromatin fraction and the expression increased 24–48 hours after HCMV infection.

## Discussion

Evidence from several studies indicates that the DNA genome of herpesviruses is devoid of any nucleosomes within the virus particle [32]–[34]. In contrast, in infected nuclei, herpesvirus genomes form structures that resemble cellular chromatin and these structures change in composition throughout the time course of infection [35]–[38]. As early as two hours post infection, a fraction of the HCMV genomes is associated with histones, but eventually, the HCMV progeny genomes have to be stripped naked before being packaged [9]. Factors involved in DNA replication, repair and transcription do not get access to DNA packed in chromatin and thus have to act in concert with chromatin modifiers and remodelers that loosen the chromatin grip on DNA. The SWI/SNF family of chromatin remodeling complex utilizes the energy of ATP hydrolysis to remodel chromatin structures, thereby participating in gene regulation, replication, viral integration, control of cell growth and tumor suppression [25], [39]. SMARCB1 was initially identified as a cellular partner to the HIV-1 integrase [40]. Subsequent studies have revealed the interaction of SMARCB1 or other subunits of the SWI/SNF complex with viral proteins from human papillomavirus [41]–[43], Epstein-Barr virus [44], Kaposi's sarcoma-associated herpes virus [45] and herpes simplex virus −1 [46], [47]. Collectively, these studies have shown that the SWI/SNF complex is crucial for effective viral gene transcription and DNA replication. In search for cellular partners of UL114, a nearly full-length SMARCB1 clone was identified as an interacting partner. The direct interaction between UL114 and SMARCB1 was validated *in vitro* by three different experiments.

UL114 has been found to interact with UL44 and this complex was highly enriched in viral replication foci [16], [26]. Since co-localization between UL114 and SMARCB1 could not be carried out, UL44 was used as a replication marker. By this mean we showed that SMARCB1 co-localizes to replication foci as early as 24 hpi. The SWI/SNF complex consists of at least nine subunits that are conserved among eukaryotes [48]. Four of the subunits; the central ATPase subunit, either hBRM (Bramha) or Bramha-related gene-1 (BRG-1), SMARCB1, BAF170 and BAF155 are required for efficient chromatin remodeling [28]. Our results demonstrate that the expression of these core subunits of the SWI/SNF chromatin remodeling complex increased during HCMV infection and were relocated to and concentrated in virus replication foci of HCMV-infected fibroblasts. To our knowledge, this is the first time a chromatin remodeling complex has been detected in virus replication foci in HCMV. Furthermore, interaction between SMARCB1 and UL44 was demonstrated both by detection of SMARCB1 in cell extracts precipitated with anti-UL44 antibodies and by direct interaction between recombinant proteins. Thus, we have evidence of direct interactions between SMARCB1 and UL114 and SMARCB1 and UL44. The UL44 and/or UL114 proteins may be of importance to recruit and stabilize the SWI/SNF chromatin remodeling complex to the replication centers.

The human HCMV DNA polymerase is composed of a catalytic subunit, UL54, which possesses basal DNA polymerase activity [49], and the accessory protein, UL44 which has been shown to specifically interact with UL54 and to stimulate long-chain DNA synthesis by UL54 [50], [51]. UL44 is a multifunctional protein capable of associating/interacting with several other viral and host proteins [16], [26], [52]–[55]. Viral replication centers also serve as foci for viral gene expression, presumably in part by concentrating templates for transcription with the proteins that carry out or regulate this process. Thus, the presence of the SWI/SNF chromatin remodeling complex in replication foci throughout infection and its association with UL114 and with UL44 might imply its involvement in different DNA transactions. For example, it has been shown that the UL44 gene product from the late viral transcript is required for efficient viral gene expression rather than viral DNA synthesis [56]. Comparable to the herpes simplex virus type-1 single-strand DNA-binding protein, ICP8, which co-precipitates with chromatin remodeling factors [47], UL44 could recruit the SWI/SNF complex to late viral promoters at late times after infection.

Finally, although controversial and ill-defined, the nuclear matrix, also referred to as nucleoskeleton or scaffold, organizes the eukaryotic DNA into topologically distinct loops. This is generated by the attachment of chromatin fibers to the nuclear matrix via specific regions called scaffold or matrix attachment regions S/MAR [57], [58]. DNA replication and transcription of active DNA are found tightly associated with the nuclear matrix, while inactive loci are not [59]–[61]. Several studies have reported replication and expression of viral genomes in association with the nuclear matrix [62]–[64]. SMARCB1 has been found to be associated with the nuclear matrix and chromatin [31]. UL44 has also been shown to be associated with the nuclear matrix [65]. By fractionating the nucleus into the sub-nuclear structures; chromatin and nuclear matrix, we showed that in addition to SMARCB1 and UL44, UL114 was present in the chromatin and nuclear matrix fraction, but highly enriched in the chromatin fraction. However, it remains to be investigated whether SMARCB1, UL114 and UL44 associate in one complex and/or as different complexes (in the nuclear matrix and/or associated with chromatin) throughout the time course of infection allowing chromatin remodeling both during DNA replication, DNA transcription and DNA packaging.

## Materials and Methods

### Cells and Virus

Human embryonic fibroblast (HE) cells were obtained from the National Institute of Public Health, Oslo, Norway. HE-cells were grown and maintained in 1∶1 minimal essential medium (MEM)+Dulbecco's modification of Eagle's medium (DMEM) (Gibco, LifeTechnologies Ltd. supplemented with endotoxin-free fetal calf serum (FCS), L-glutamine (0.3 mg/ml), gentamicin (40 µg/ml), amphotericin B (Fungizone) (2.5 µg/ml), and penicillin G (6 µg/ml). Medium with 10% FCS was used for propagation of the cells whereas medium with 2% FCS was used for maintenance of the cells. The HE-cells were routinely screened for mycoplasma by DNA staining with MycoAlert Mycoplasma Detection kit (Lonza Rockland Inc.).

Stocks of highly purified human cytomegalovirus (HCMV) laboratory type strain AD169 (American Type Culture Collection (ATCC), Rockville, MD) was propagated in low passage HE cells. Virus was propagated at low virus to cell ratios to minimize generation of defective particles and purified as described previously [66].

### Cell cycle synchronization and infections

All cell experiments were performed subsequent to release from contact inhibition. The cells were grown to confluence, and after three days of confluence, they were trypsinized and re-plated at a lower density (10^6^cells/75 cm^2^) to induce progression into the cell cycle. At the time of re-plating (1∶1 MEM+DMEM-10% FCS), the cells were infected with HCMV at a multiplicity of infection (moi) of 5 plaque forming unit (PFU) per cell or mock infected. Virus adsorption was allowed for one hour before the medium was discarded and fresh medium (1∶1 MEM+DMEM-10% FCS) was added. Only experiments where more than 95% of the cells were infected were accepted. The infection was assessed at 24 hpi by an immunocytochemical method employing E13 monoclonal antibodies specific for IE1 and 2 antigens (Seralab UK).

At different time-points post infections, the cells were scraped off for preparation of cell lysates or trypsinized for preparation of nuclear extract or cytospins for immunostaining.

### Preparation of cell lysates and nuclear extracts

Cells for preparation of cell lysates were harvested by scraping cells into ice-cold PBS, followed by centrifugation at 1000× g for 4 minutes at 4°C. Cell pellets were lysed using a modified RIPA buffer (50 mM Tris-HCL, pH 7,4, 150 mM NaCl, 1 mM EDTA, 1% NP-40 (Igepal CA-630, Sigma-Aldrich), 1 mM PMSF and Protease Inhibitor Cocktail (PIC) (P 8340, Sigma-Aldrich), and incubated at 4°C for 15 minutes, followed by centrifugation at 14000× g for 15 minutes at 4°C . The supernatants were decanted into a fresh tube and stored at −70°C until used. Nuclear extracts were made by plasmolysis of mock or HCMV-infected (5 PFU/cell) fibroblast as previously described [66], [67].

### Nuclear matrix preparation

Nuclear matrix proteins were fractionated from the indicated cells according to the method of He and collaborators (1990) [68]. Cells were washed twice in PBS and treated with 500 µl of CSK buffer (10 mM PIPES pH 6.8, 100 mM NaCl, 300 mM sucrose, 3 mM MgCl_2_, 1 mM EDTA, 1 µg/ml leupeptin and pepstatin, 1 mM PMSF and 0.1% Triton X-100) for 10 min on ice. The cells were collected and centrifuged at 5000 *g* for 2 min. The soluble cytoplasmic fraction was removed and the pellet resuspended in 200 µl of CSK buffer containing 100 U RNase-free DNase I (Roche Diagnostics). After 15 min at 37°C, ammonium sulfate was added to a final concentration of 0.25 M. The samples were rotated 5 min at room temperature and centrifuged as above. The soluble chromatin fraction was removed, the pellet washed in CSK buffer with 2 M NaCl for 5 min at 4°C and centrifuged as above. The supernatant was removed and the nuclear matrix pellet resuspended in 100 µl 3× Laemmli buffer and equal cell equivalents from each fraction were subjected to conventional western blot analyses.

### Immunoprecipitation

750 µg of either mock or HCMV lysates incubated with 2.5 µg of rabbit polyclonal SMARCB1 antibody (a kind gift from Dr. Imbalzano, University of Massachusetts Medical School, US) or 750 µg of HCMV lysate without antibody were incubated for 1 h at 4°C with gentle agitation. Immunocomplexes were precipitated by the addition of Protein G beads (GE Healthcare Bio-Science AB) and incubated for 2 h at 4°C. After sufficient washing with modified RIPA buffer the immunoprecipitate was eluted in 1× Laemmli NuPage buffer by preheating to 70°C for 20 min and separated by 10% Nupage SDS-PAGE and further subjected to conventional western blot analysis.

### Western blotting

Proteins were separated by using the NuPage system (10% gel) (Invitrogen) as described by the manufacturer and electroblotted to a polyvinylidene fluoride membrane (Immobilon-P/Immobilon-FL, Millipore). Primary antibodies used were mouse anti-GST ((3D4), sc-57753, Santa Cruz Biotechnology), goat anti-lamin A/C antibody (N-18, Santa Cruz Biotechnology), rabbit anti-histone H1 antibody (FL-219, Santa Cruz Biotechnology), mouse anti-UL57 ((anti-cytomegalovirus ICP8, CH167), ab-53493 Abcam), rabbit anti-UL114 (polyclonal ascites/serum raised against a synthetic peptide comprising amino-acids 11–28+cys of UL114), mouse anti-UL44 (cat. No ABV006, Autogenbioclear), rabbit anti-SMARCB1 (ab12167, Abcam), mouse anti-GAPDH (cat. no. 4300, Ambion), mouse anti-BRG-1 ((G7), sc-17796 Santa Cruz Biotechnology), mouse anti-BAF155 ((DXD7), sc-32763 Santa Cruz Biotechnology) and mouse anti-BAF170 ((E6), sc-17838 Santa Cruz Biotechnology). Secondary antibodies were peroxidase-conjugated anti-mouse or anti-rabbit IgG antibodies (Promega). Membranes were visualized with the use of ECF-PLUS (Amersham) and PhosphoImager (Molecular Dynamics).

### Immunofluorescence microscopy

Cytospins with 10^5^ cells were fixed with ice-cold methanol/acetone (1∶1) for 90 s and frozen at −70°C until use. Cytospins were washed in TBS before incubation with primary and secondary antibodies diluted in serum diluent (TBS with 12.5% HCMV-negative serum). The cytospins were incubated at 4°C over night with the primary antibodies; mouse monoclonal anti-UL44 (2 µg/ml) (CA006-100, Virusys Corp.), rabbit anti-SMARCB1 (5 µg/ml) (ab12617, Abcam), mouse anti-BAF155 (DXD7) (0,4 µg/ml) (sc-32763 Santa Cruz Biotechnology Inc.), mouse anti-BAF170 (E-6) (0,4 µg/ml) (sc-17838 Santa Cruz Biotechnology Inc.), mouse anti-BRG-1 (G-7) (0,4 µg/ml) (sc-17796 Santa Cruz Biotechnology Inc.) before incubation with secondary antibodies, anti-rabbit Alexa Fluor® 594 f(ab′)_2_ (Molecular Probes A11072) and anti-mouse Alexa Fluor® 488 f(ab′)_2_ (Molecular Probes A11070), for 1 hour at room temperature. The cover slips were mounted in Vectashield medium containing Dapi (1.5 µg/ml) (Vector Laboratories) and analyzed using a Zeiss LSM 510 META confocal microscope (63×/1.4 NA Plan-Apochromat oil-immersion objective) with the following settings: DAPI; Blue diode 405 laser/band-pass filter 420–480 nm, Alexa488; Argon–ion 488 laser/long-pass filter 505 nm, Alexa 594; and HeNe 543 laser/long-pass filter 560 nm. Sections (2.5–3 µm thick) were optically sliced (optical slice thickness of ∼0.8 um) into 8–10 images that were projected on top of one another to give the images presented.

### Plasmids

The UL114 DNA sequence was PCR-amplified for cloning into the *ECORI* site of the two-hybrid vector pGBKT7 (MATCHMAKER two-hybrid systems, Clontech) with primers: 5′- CGG AAT TCA TGG CCC TCA AGC AGT GGA TG-3′ (forward) and 5′- CCC CGA ATT CAC CCA CAG AGT CGC CA-3 (reverse) and pDEST14 –UL114 [26] as a template. All clones were confirmed by sequencing the insert on both strands.

### Yeast Two-hybrid Screening

The Clontech GAL4 MATCHMAKER yeast two-hybrid system was used according to the manufacturer's (Clontech) instructions. The Pretransformed MATCHMAKER brain cDNA library (PT3183-1) were precloned into a yeast GAL4 activation domain (AD) vector (pACT2, *LEU2*+), pretransformed into *Saccharomyces cerevisiae* host strain Y187 (*MAT*α, *MEL1*, *lacZ*) and used to screen for binding partners for UL114 cloned into pGBKT7 vector in frame to the DNA binding domain (construct pGBKT7 -UL114) in the yeast strain *Saccharomyces cerevisiae* host strain AH109 (*MAT*a, *HIS3*, *ADE2*, *MEL1*, lacZ).

### Coupled *in vitro* transcription and translation and co-immunoprecipitation

The GAL4 activation vector (AD) (pACT2) used for constructing pretransformed libraries lacked both a T7 RNA polymerase promoter and an epitope tag. By using PCR with appropriate primers (as indicated by MATCHMAKER Co-IP kit, BD Bioscience) we introduced T7 and HA tag sequences upstream of the collected library cDNA while amplifying the insert for *in vitro* transcription and translation. *In vitro* transcription/translation was carried out using the T_N_T® Quick Coupled transcription/translation system (Promega Corp.) to synthesis ^35^S labeled and tagged protein according to the manufacturer's instructions.

Following translation, co-immunoprecipitation according to the manufacturer's instructions (Matchmaker Co-IP kit, PT3323-1, BD Bioscience) was carried out followed by conventional SDS-PAGE (8% SDS–polyacrylamide) and PhoshorImager (Molecular Dynamics).

### Expression and purification of recombinant proteins

Expression and purification of UL114, His_6_-UL44 and His_6_-NEIL1 (hFPG1) were carried out as described previously [26], [69]. Human full-length SMARCB1 (Ultimate™ ORF Clone IOH29630, pENTR™221 Invitrogen) which are compatible with the *Escherichia coli (E.coli)* Expression System Gateway Technology was transferred into pDEST15 (GST-tag at the N-terminal site) by recombination according to the manufacturer's protocols. *E.coli* BL21-AI™ cells were transformed with pDEST15-SMARCB1 plasmid. Cells were grown at 37°C in 3 L of LB^amp^ medium containing D-Sorbitol and Betaine to an absorbance of approximately 0.3 at 600 nm. Expression was induced by addition of L-arabinose to a final concentration of 0.2%. Cells were grown for an additional 16 hours at 18°C and then centrifuged at 6000 g for 20 min and stored at −20°C before use. The thawed cell pellet was resuspended in 45 mL PSB buffer containing 5 mM DTT, and the cells were lysed by sonication. Next, the lysate was centrifuged at 13000 rpm for 30 min at 4°C. The supernatant was applied directly to a 5 mL GStrap column equilibrated in PBS containing 5 mM DTT and the tagged protein was eluted in 50 mM Tris-HCl, pH 8.0 containing 10 mM reduced glutathione and 10 mM DTT. Fractions containing purified protein were concentrated and further purified by gel filtration chromatography (superdex75 equilibrated in 10 mM Tris-HCl pH 7.5, 50 mM NaCl and 10 mM β-ME).

### Expression and purification of crude GST extract

*E.coli* BL21 codon Plus cells (BL21-CodonPlus (DE3)-RIL strain, cat. nr. 230245, Stratagene) were transformed with pGEX-3X (Code no: 27-4803-01, Pharmacia Biotech) to express crude GST. The transformed cells were grown at 37°C in 0.5 L LB medium containing 100 µg/ml ampicillin and expression of GST was induced with 1 mM isopropyl-ß-δ-thiogalactopyranoside (IPTG) at OD_600_ of 0.7. The cells were harvested after four hours. The cell pellet was resuspended in PBS with 1 mM DTT (5 ml per g cell pellet) and cell-free extract was prepared by sonication and centrifugation (14000 g) for 30 min.

### GST pull-down assay

Crude GST-extract and purified GST-SMARCB1 were subjected to GST pull-down analysis with the Rapid purification of GST-Fusion Proteins kit (Cat 3 MG-101, Bioclone Inc) using the BcMag®GST magnetic beads according to the manufacturer's protocol with some modifications. Briefly, 30 µl BcMag beads were incubated with 10 µg purified recombinant GST-SMARCB1 or 50 µg crude GST-extract (negative control) for 30 min at room temperature. Next, 20 µg purified recombinant UL114 or 3 mg of modified RIPA lysate from mock or HCMV infected cells harvested at 72 hpi were added to the BcMag®GST magnetic beads. After washing in GST Binding/Washing buffer the co-precipitate was eluted in NuPage loading buffer by heating at 70°C for 20 min. The proteins were separated by SDS-PAGE and verified by western blot analysis.

### His pull-down assay

Purified His_6_-UL44, His_6_-NEIL1, GST-SMARCB1 and GST were subjected to conventional his-tag pull-down analysis with Dynabeads® His-Tag Isolation & Pull-down (Cat. No 101.03D) according to the manufacturer's protocol (Dynal, Oslo, Norway) with some modifications. Briefly, 12.5 µl Dynabeads were incubated with 15 µg His_6_-UL44 or His_6_-NEIL1 in binding-buffer (50 mM Na-Phosphate, pH 8.0, 600 mM NaCl, 50 mM Imidazole, 0.01% Tween-20). Next, the Dynabeads were incubated with GST-SMARCB1 (20 µg) or GST (20 µg) in Pull-down buffer ( 6.5 mM Na-phosphate, pH 7.4, 140 mM NaCl, 50 mM Imidazole, 0.01% Tween-20). Finally, the Dynabeads were incubated with 60 µl His Elution buffer (300 mM Imidazole, 50 mM Na-phosphate pH 8.0, 300 mM NaCl, 0.01% Tween-20). The eluted proteins were analyzed by SDS-PAGE.

## Supporting Information

Figure S1 ***In vitro*** **binding analysis of HA-tagged clone 4 and c-myc-tagged UL114 in ^35^S-labeled proteins using the TNT coupled transcription/translation system.** The proteins were transcribed and translated *in vitro* with ^35^S-methionine in the translation mixture to generate radioactive labeled products from vectors pACT2-clone 4 (HA-epitope) and pGBKT7-UL114 (c-myc epitope). The translated clone 4-HA and UL114-c-myc were immunoprecipitated with either anti-HA or anti-c-myc-antibodies, eluted from the Protein G beads and immunoprecipitates (10 µl) were subjected to 8% SDS-PAGE and PhosphoImaging. Lane 1: UL114-c-myc+c-myc antibody. Lane 2: clone 4-HA+HA-antibody. Lane 3: clone 4-HA+UL114-c-myc+HA-antibody. Lane 4: clone 4+UL114-c-myc+c-myc antibody.
(TIF)

Figure S2 **Mock control for the antibodies UL44 and SMARCB1 used in the co-localization studies of UL44 and SMARCB1 in HCMV-infected fibroblast cells harvested at 24, 48, and 72 hpi.** The cells were fixed and subjected to double-staining for UL44 (mouse Mab-UL44) and SMARCB1 (rabbit Pab-SMARCB1) for immunofluorescence microscopy. Secondary antibodies used for staining were: UL44 in green (anti-mouse 488) and SMARCB1 in red (anti-rabbit 594), and cells were visualized by confocal microscopy. Co-localization was visualized by a merge of the two microscopic determinations, and counterstaining of the nuclei was achieved by the use of DAPI.
(TIF)

Figure S3 **Control for the interaction between SMARCB1 and UL44 using His_6_-NEIL1 as an irrelevant protein.** *In vitro* pull-down assay of GST-SMARCB1 and His_6_-NEIL1. Purified GST-SMARCB1 (20 µg) or GST (20 µg) incubated with purified His_6_-NEIL1 (15 µg) immobilized on magnetic His-tag Dynabeads. Samples were analyzed by SDS-PAGE and Coomassie blue staining. Lane 1: GST-SMARCB1+Dynabeads His-tag. Lane 2: His_6_-NEIL1+Dynabeads His-tag. Lane 3: GST-SMARCB1+His_6_-NEIL1+Dynabeads His-tag. Lane 4: GST+His_6_-NEIL1+Dynabeads His-tag. Lane 5: GST (input, 2 µg, 10%). Lane 6: GST-SMARCB1 (input, 2 µg, 10%). Lane 7: His_6_-NEIL1 (input, 2 µg, 13%). Note that spontaneous cleavage occurred in the GST-SMARCB1 protein sample (Lane 5).
(TIF)
