# Supplementary figures and images for: The Chromatin Remodeling Factor SMARCB1 Forms a Complex with Human Cytomegalovirus Proteins UL114 and UL44
Source: PLoS One. 2012 Mar 27;7(3):e34119. doi: 10.1371/journal.pone.0034119 (PMC3313996; doi:10.1371/journal.pone.0034119)

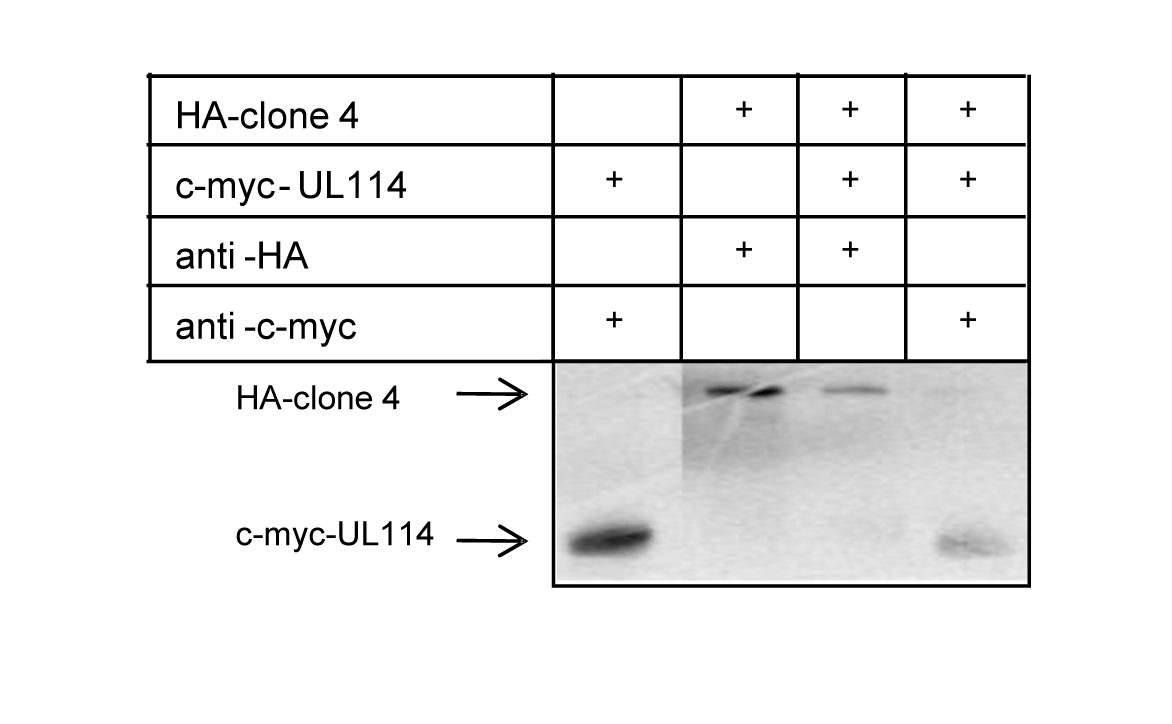

Supplement: Figure S1 — In vitro binding analysis of HA-tagged clone 4 and c-myc-tagged UL114 in 35S-labeled proteins using the TNT coupled transcription/translation system. The proteins were transcribed and translated in vitro with 35S-methionine in the translation mixture to generate radioactive labeled products from vectors pACT2-clone 4 (HA-epitope) and pGBKT7-UL114 (c-myc epitope). The translated clone 4-HA and UL114-c-myc were immunoprecipitated with either anti-HA or anti-c-myc-antibodies, eluted from the Protein G beads and immunoprecipitates (10 µl) were subjected to 8% SDS-PAGE and PhosphoImaging. Lane 1: UL114-c-myc+c-myc antibody. Lane 2: clone 4-HA+HA-antibody. Lane 3: clone 4-HA+UL114-c-myc+HA-antibody. Lane 4: clone 4+UL114-c-myc+c-myc antibody. (TIF) [file pone.0034119.s001.tif]

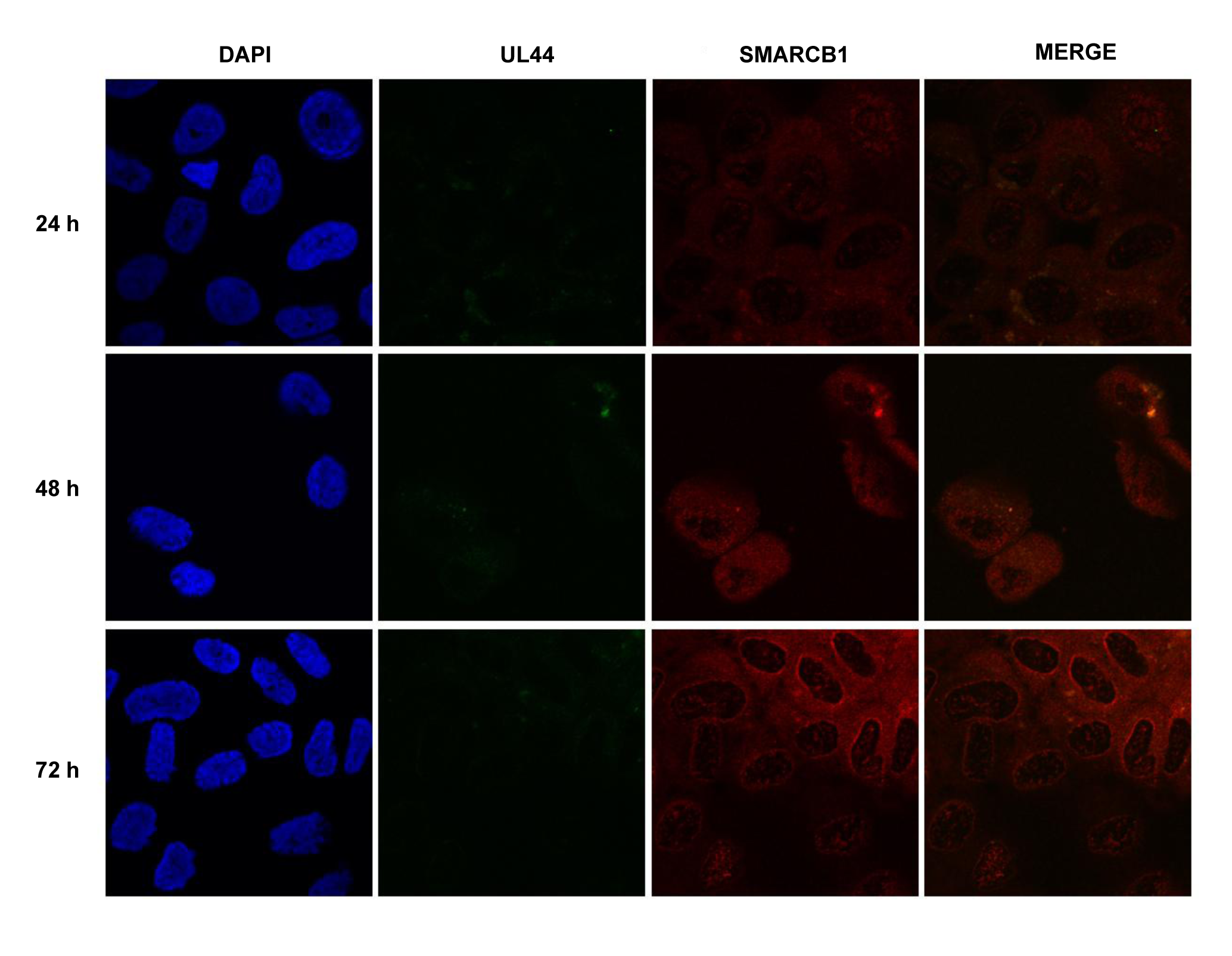

Supplement: Figure S2 — Mock control for the antibodies UL44 and SMARCB1 used in the co-localization studies of UL44 and SMARCB1 in HCMV-infected fibroblast cells harvested at 24, 48, and 72 hpi. The cells were fixed and subjected to double-staining for UL44 (mouse Mab-UL44) and SMARCB1 (rabbit Pab-SMARCB1) for immunofluorescence microscopy. Secondary antibodies used for staining were: UL44 in green (anti-mouse 488) and SMARCB1 in red (anti-rabbit 594), and cells were visualized by confocal microscopy. Co-localization was visualized by a merge of the two microscopic determinations, and counterstaining of the nuclei was achieved by the use of DAPI. (TIF) [file pone.0034119.s002.tif]

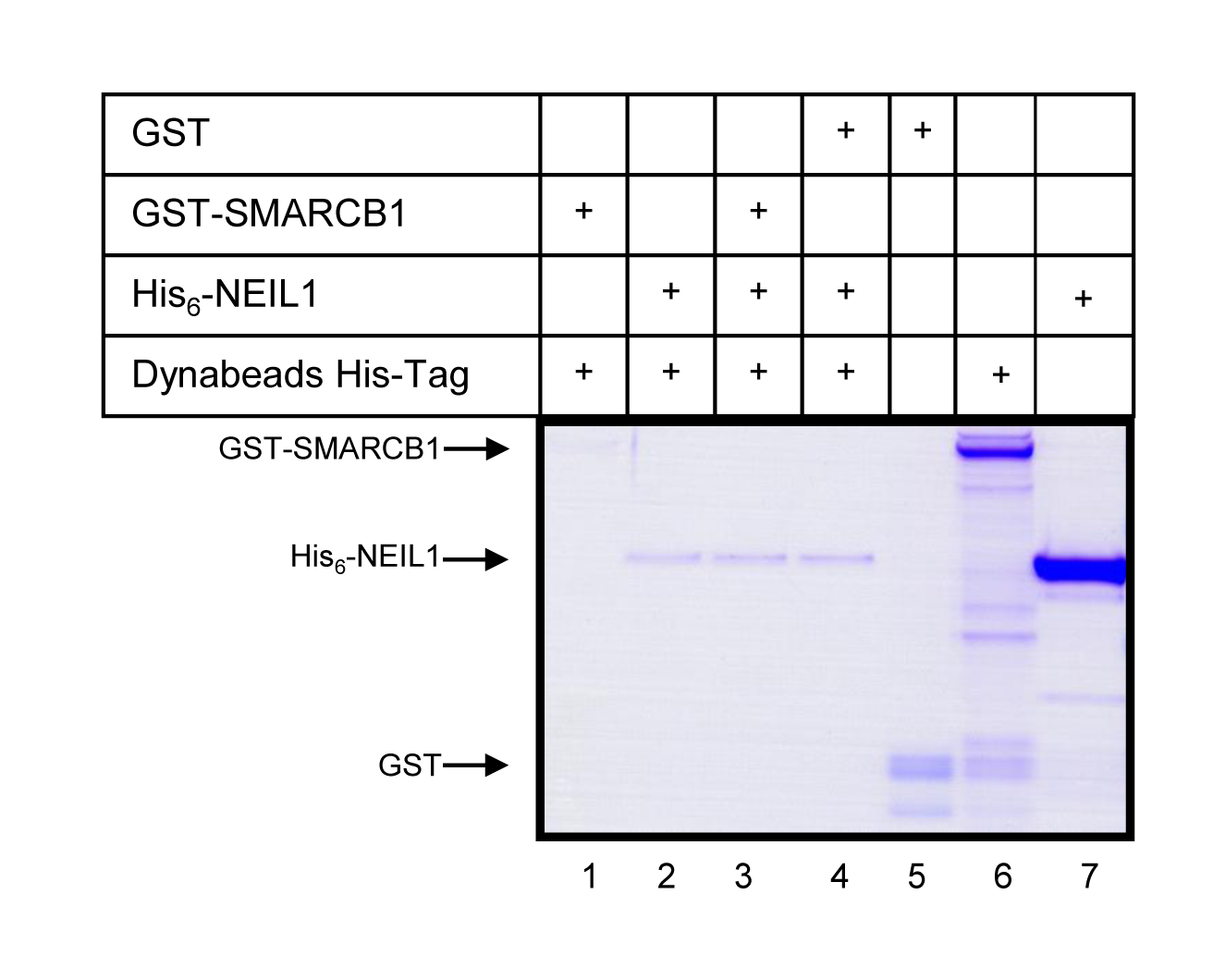

Supplement: Figure S3 — Control for the interaction between SMARCB1 and UL44 using His6-NEIL1 as an irrelevant protein. In vitro pull-down assay of GST-SMARCB1 and His6-NEIL1. Purified GST-SMARCB1 (20 µg) or GST (20 µg) incubated with purified His6-NEIL1 (15 µg) immobilized on magnetic His-tag Dynabeads. Samples were analyzed by SDS-PAGE and Coomassie blue staining. Lane 1: GST-SMARCB1+Dynabeads His-tag. Lane 2: His6-NEIL1+Dynabeads His-tag. Lane 3: GST-SMARCB1+His6-NEIL1+Dynabeads His-tag. Lane 4: GST+His6-NEIL1+Dynabeads His-tag. Lane 5: GST (input, 2 µg, 10%). Lane 6: GST-SMARCB1 (input, 2 µg, 10%). Lane 7: His6-NEIL1 (input, 2 µg, 13%). Note that spontaneous cleavage occurred in the GST-SMARCB1 protein sample (Lane 5). (TIF) [file pone.0034119.s003.tif]
